# NOTCH signalling – a core regulator of bile duct disease?

**DOI:** 10.1242/dmm.050231

**Published:** 2023-08-22

**Authors:** Anabel Martinez Lyons, Luke Boulter

**Affiliations:** ^1^ MRC Human Genetics Unit, Institute of Genetics and Cancer, Edinburgh EH4 2XU, UK; ^2^ CRUK Scottish Centre, Institute of Genetics and Cancer, Edinburgh EH4 2XU, UK

**Keywords:** Bile duct, Cancer, Liver, NOTCH

## Abstract

The Notch signalling pathway is an evolutionarily conserved mechanism of cell–cell communication that mediates cellular proliferation, fate determination and maintenance of stem/progenitor cell populations across tissues. Although it was originally identified as a critical regulator of embryonic liver development, NOTCH signalling activation has been associated with the pathogenesis of a number of paediatric and adult liver diseases. It remains unclear, however, what role NOTCH actually plays in these pathophysiological processes and whether NOTCH activity represents the reactivation of a conserved developmental programme that is essential for adult tissue repair. In this Review, we explore the concepts that NOTCH signalling reactivation in the biliary epithelium is a reiterative and essential response to bile duct damage and that, in disease contexts in which biliary epithelial cells need to be regenerated, NOTCH signalling supports ductular regrowth. Furthermore, we evaluate the recent literature on NOTCH signalling as a critical factor in progenitor-mediated hepatocyte regeneration, which indicates that the mitogenic role for NOTCH signalling in biliary epithelial cell proliferation has also been co-opted to support other forms of epithelial regeneration in the adult liver.

## Teaser

**Summary:** Unconstrained tissue repair can lead to cancer. We explore how NOTCH signalling promotes ductular regeneration while asking whether it can be inhibited to limit tumour growth.

## Introduction

A number of adult tissues rely on the formation of biological tubes to function. In the vertebrate liver, these tubular structures are known as bile ducts and are composed of specialized and heterogeneous biliary epithelial cells (BECs), which modify bile and allow the hepatic secretion of metabolic byproducts (Pepe-Mooney et al., 2019). Although BECs only constitute approximately one-fifth of all epithelial cells in the liver, BECs are crucial for normal hepatic function. In patients with chronic biliary disease in which the form or function of the bile ducts is compromised, liver transplantation is often the only therapeutic option (Banales et al., 2019; Ferreira-Gonzalez et al., 2018).

The mechanisms that govern the regrowth or repair of intrahepatic bile ducts in the adult are not well characterized. It has become clear, however, that as we understand more about embryonic ductular development, many of the essential signals for biliary ontogeny are reactivated following injury and play a role in ductular regeneration and pathogenesis in the postnatal organ (Boulter et al., 2012; Jung et al., 2008; Wilson et al., 2020).

In this Review, we focus on NOTCH signalling as a paradigm of reiteratively used signals that are critical for normal bile duct development (this has been extensively reviewed elsewhere; Martinez Lyons and Boulter, 2021). We then explore how re-expression of NOTCH and its activation is a master regulator of biliary biology in the context of adult biliary disease and cancer.

## The Notch signalling pathway as a coordinator of cell–cell communication

**Fig. 1.**
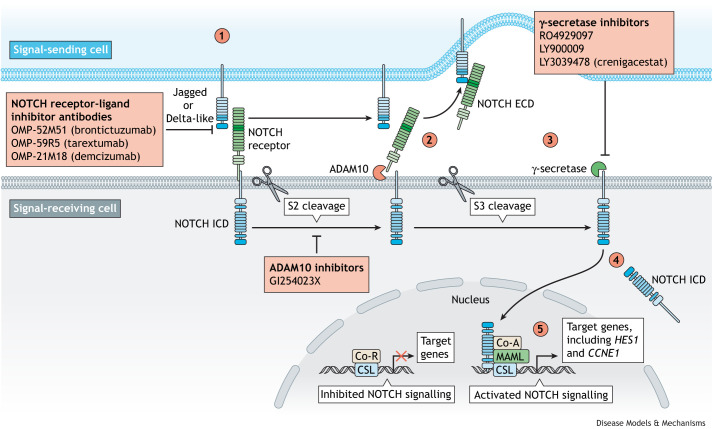
**NOTCH, a major regulator of cell state.** The NOTCH signalling cascade is a major regulator of cell–cell communication in multicellular organisms. In order for adjacent cells to communicate, (1) NOTCH receptors interact with their cognate ligands, Jagged (JAG1 and JAG2) or Delta-like (DLL1, DLL3 and DLL4). (2) Following receptor–ligand interaction, conformational changes in the NOTCH receptor make it permissive to cleavage through ADAM10 or ADAM17 (S2 cleavage). (3) The presenilin-containing complex, γ-secretase, then cleaves the residual NOTCH receptor (S3 cleavage). (4) Following this, the NOTCH intracellular domain (ICD) translocates to the nucleus. (5) In the nucleus, the NOTCH ICD interacts with a transcriptional activation protein complex (Co-A), mediated by C promoter-binding factor 1/Suppressor of Hairless/LIN-12 and GLP-1 (CSL; also called RBPJκ in mice). This interaction displaces a histone deacetylase transcriptional repressor complex (Co-R), thus permitting the expression of a number of target genes. Pharmacological inhibition of NOTCH signalling at a number of steps in the pathway, including receptor–ligand interactions, ADAM10-dependent receptor processing and γ-secretase-dependent cleavage, downregulates pathway activation. ECD, extracellular domain.

The Notch signalling pathway (Fig. 1) is a conserved mechanism of short-range, cell–cell communication that mediates a number of essential processes, including cellular proliferation and survival, cell fate specification and apoptosis (Artavanis-Tsakonas et al., 1999; Kopan and Ilagan, 2009; Milán et al., 2002). In addition to being essential for embryonic development (Yu et al., 2008; Zhu et al., 2006), growing evidence has shown that Notch signalling also regulates the postnatal homeostasis of adult tissue stem cells (Liu et al., 2010; Sato et al., 2012).

The mammalian NOTCH signalling pathway consists of four NOTCH receptors (NOTCH1-4), five canonical ligands [jagged (JAG)1 and JAG2, and delta-like (DLL)1, DLL3 and DLL4] and numerous auxiliary factors that transduce and regulate the signal (Artavanis-Tsakonas et al., 1999; Kopan, 2012). In canonical NOTCH signalling, binding of the extracellular domain (ECD) of a NOTCH receptor to the extracellular region of a corresponding ligand on a neighbouring cell triggers proteolytic cleavage of the receptor.

The NOTCH ECD and bound ligand are then endocytosed by the signal-sending cell, a process primarily mediated by the E3 ubiquitin ligase MIB-1 and the Epsin ubiquitin-binding adaptor proteins (Le Borgne et al., 2005; Wang and Struhl, 2005). Meanwhile, on the signal-receiving cell, the NOTCH receptor undergoes further proteolytic cleavage by γ-secretase (De Strooper et al., 1999), an enzymatic complex that contains presenilin, nicastrin, presenilin enhancer 2 and Anterior pharynx-defective 1 proteins (Bray, 2006; Fortini, 2002; Kopan, 2012). γ-secretase-mediated cleavage releases the NOTCH intracellular domain (ICD) (Hori et al., 2013), the only direct messenger in the pathway. The NOTCH ICD translocates to the nucleus, where it interacts with DNA-binding transcriptional co-activators CSL (also called RBPJκ in mice) and MAML to displace a CSL-bound transcriptional repressor complex (Borggrefe and Oswald, 2009; Bray, 2006). Upon interaction with the NOTCH ICD, CSL becomes a potent transcriptional activator of target genes, most commonly those belonging to the Hairy and Enhancer-of-split families, HES and HEY (reviewed in Fischer and Gessler, 2007).

## Learning from development

To fully appreciate the role of NOTCH signalling in adult biliary homeostasis and disease, it is important to outline its essential involvement in biliary development. In the embryonic liver, bile ducts form as a tubular network that arises from a transient structure known as the ductal plate. The ductal plate is specified from a bipotent progenitor population called hepatoblasts, and undergoes a series of complex morphogenic rearrangements to form a functional tubular network with a contiguous structure and a continuous lumen (Antoniou et al., 2009; Tanimizu et al., 2016).

The initial indication that NOTCH signalling is necessary for the formation of bile ducts came from classical human genetic studies in children born with Alagille syndrome (ALGS) (Alagille et al., 1975; Li et al., 1997), a multisystemic disorder in which affected individuals fail to form bile ducts within the liver, known as bile duct paucity (Alagille et al., 1987). Approximately 95% of ALGS patients carry a single causal mutation within the NOTCH ligand gene, *JAG1*; however, less frequently, hypomorphic mutations in *NOTCH2* also result in ALGS (McDaniell et al., 2006).

A number of studies in model organisms have recapitulated the ALGS phenotype. Work in zebrafish has demonstrated that endodermal expression of *jagged 2b* (*jag2b*), an ortholog of human *JAG2*, is essential for the formation of ducts in the liver and pancreas (Lorent et al., 2004; Zhang et al., 2017), and that its loss results in a failure to form bile ducts. Furthermore, mice that are doubly heterozygous for loss of JAG1 and a hypomorphic variant of NOTCH2 exhibit the characteristic loss of intrahepatic bile ducts observed in ALGS (McCright et al., 2002).

Studies demonstrating the centrality of NOTCH signalling in bile duct development and in patterning of the vertebrate biliary system have been reviewed previously (Martinez Lyons and Boulter, 2021). What remains poorly understood is how this essential signal orchestrates bile duct pathophysiology and pathogenesis in postnatal life.

## Reactivation of the NOTCH programme during progenitor-mediated hepatocyte regeneration

The adult liver has a remarkable regenerative capability. Following injury, mature, terminally differentiated cells exit quiescence and proliferate to reconstitute damaged tissue. Indeed, hepatocytes in the liver are able to proliferate such that, in mice, 70% of the liver can be removed and the normal mass will be recovered within a matter of weeks (reviewed in Michalopoulos, 2017). In chronic disease, in which the capacity of mature epithelial cells to regenerate is overwhelmed or inhibited, a population of facultative tissue-specific hepatic progenitor cells (HPCs; also known as reactive cholangiocytes, oval cells, liver progenitor cells and, recently, transitional liver progenitor cells) differentiate into mature epithelial cell types. There may be subtle, functional differences between these cell populations, and it is clear from recent single-cell RNA-sequencing studies that there is some heterogeneity within this progenitor pool (Pepe-Mooney et al., 2019; Planas-Paz et al., 2019; Pu et al., 2023). However, these descriptive terms are often used interchangeably to describe cells of a bile duct origin that proliferate in response to damage and in certain contexts undergo differentiation into hepatocytes. It is widely accepted then that the differentiated progeny of HPCs integrate into tissue structures and contribute to the restoration of normal liver function (Aloia et al., 2019; Gadd et al., 2020; Raven et al., 2017).

HPCs are largely absent in the healthy liver, but infrequent HPCs or a precursor population are believed to reside within the canals of Hering, in terminal ductules of the biliary tree (Roskams et al., 2004; Sell, 2001), and in the peribiliary glands located within the walls of larger bile ducts (Cardinale et al., 2012). Upon injury, these resident HPCs proliferate prior to terminally differentiating into mature cell types (Oertel et al., 2008; Raven et al., 2017; Yovchev et al., 2008). NOTCH signalling is a potent mitogenic signal across several tissues. In the liver, a number of studies have indicated that activation of the NOTCH signalling pathway promotes the proliferation of BECs and HPCs. Early work using a chemical bile duct injury model in mice indicated that NOTCH receptors are cleaved and the NOTCH ICD translocates to the nucleus, where it promotes proliferation of injured BECs/HPCs (Boulter et al., 2012; Jörs et al., 2015). Furthermore, chemical inhibition of this signal reduced the number of BECs that formed following injury (Boulter et al., 2012).

**Fig. 2.**
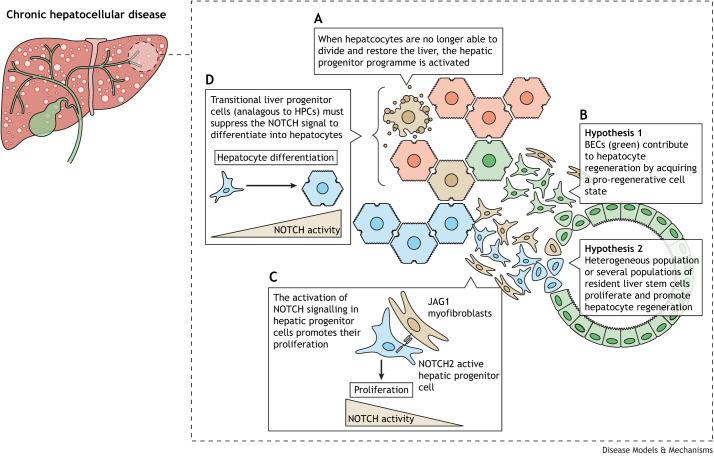
**NOTCH is required for the proliferation of hepatic progenitor cells during hepatocellular regeneration.** (A) In pathological contexts in which large-scale hepatocyte injury or senescence occurs, and in which hepatocytes can no longer regenerate the parenchyma by proliferation, a population of progenitor cells arises in the liver. (B) It is widely hypothesized that either biliary epithelial cells (BECs) demonstrate lineage plasticity and enter a pro-regenerative cell state, or that there are populations of bona fide (yet poorly characterized) stem cells within the biliary tree capable of hepatocyte regeneration. (C) Progenitor cells migrate out of the normal biliary architecture and proliferate within the parenchyma. These cells are surrounded by a complex fibroimmune microenvironment, termed the regenerative niche or ductular reaction, that provides NOTCH ligands to the progenitor cells. Signalling through NOTCH promotes the proliferation of these progenitor cells. (D) Over time, progenitor cells differentiate into mature hepatocytes and reconstitute the parenchymal hepatocytes of the liver, a process that requires the suppression of NOTCH signalling.

A limitation of these early chemical models of HPC-mediated biliary regeneration, however, was that they typically caused cumulative hepatic injury and initiated both HPC and BEC proliferation simultaneously. As such, it was unclear whether NOTCH signalling was required specifically for the proliferation and expansion of HPCs prior to differentiating into hepatocytes, which constitute the liver parenchyma. As murine models of hepatocyte regeneration have improved, it has become possible to unpick the requirement for NOTCH signalling for HPC-mediated regeneration of the hepatocyte parenchyma, and a NOTCH–IGF signalling axis has been proposed to be important in the proliferation of HPCs prior to their differentiation into hepatocytes following hepatic injury in mice (Minnis-Lyons et al., 2021). Interestingly, subsequent studies have suggested that the suppression of NOTCH signalling is required for transitional liver progenitor cells (TLPCs; an intermediate state between BECs and hepatocytes) to differentiate into hepatocytes and that sustained NOTCH activation results in the proliferation of BECs at the cost of TLPC formation (Pu et al., 2023). These two studies highlight the unknown complexity of the role of NOTCH signalling in BEC and HPC conversion into hepatocytes and reiterate the need for a careful dissection of both the epithelial heterogeneity and the role of NOTCH signalling in progenitor-mediated liver repair (Fig. 2).

## The role of NOTCH signalling in biliary disease

**Fig. 3.**
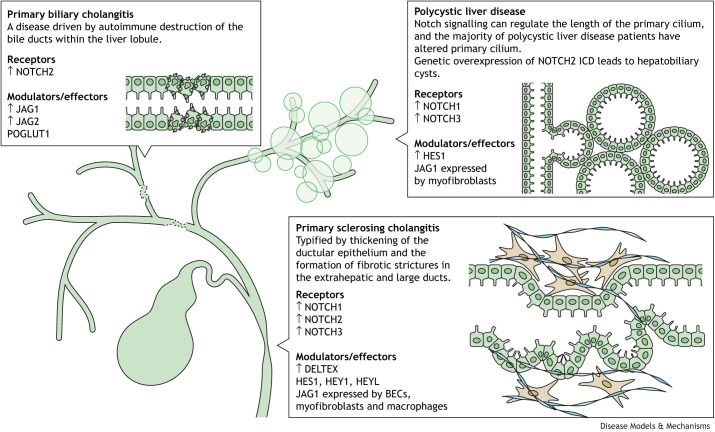
**NOTCH signalling and biliary disease: similar, but not the same.** Diverse biliary pathologies reiteratively rely on the NOTCH signalling pathway as part of their molecular pathology. Despite this, there is variation within the system, and a number of studies discussed throughout this Review have explored how different NOTCH receptors, ligands and effector proteins, such as those that modulate the post-translational modifications of NOTCH, integrate into diverse biliary disease responses. BEC, biliary epithelial cell; ICD, intracellular domain.

Adult bile duct regeneration requires the concerted involvement of epithelial, mesenchymal and inflammatory cells to restore the structure and associated function of bile ducts (Forbes and Newsome, 2016). The normal processes of ductular regeneration, however, have been found to be co-opted in a number of inherited or acquired biliary disorders that affect the hepatic bile ducts, together termed cholangiopathies (Banales et al., 2019; Fabris et al., 2017). In both inherited and acquired cholangiopathies, a pathological regenerative state leads to abnormal tubular architecture, deposition of extensive scar tissue surrounding the injured bile ducts and liver tissue (hepatobiliary fibrosis), formation of cysts and/or tumorigenesis (Fabris et al., 2017; Sneddon and Werb, 2007). Here, we discuss three cholangiopathies in which aberrant NOTCH signalling correlates with structural abnormalities and associated dysfunction of the biliary network in the adult liver (summarized in Fig. 3).

### Primary sclerosing cholangitis

Primary sclerosing cholangitis (PSC) is a chronic inflammatory disease that is characterized by diffuse fibrosis and obliterative cholangitis, which presents as strictures of the intrahepatic and extrahepatic bile ducts (reviewed in Assis and Bowlus, 2023; Karlsen et al., 2017; Sarcognato et al., 2021). The prevalence of PSC is rare, affecting ∼1 to 16 per 100,000 people in North America, with similar prevalence in the United Kingdom and throughout mainland Europe (Trivedi and Hirschfield, 2021; Trivedi et al., 2021). PSC predominantly affects males in their fourth to fifth decade of life (Hirschfield et al., 2013), and 70% of PSC patients also have a form of inflammatory bowel disease, particularly ulcerative colitis (Lunder et al., 2016; Weismüller et al., 2017). Critically, there are substantial socioeconomic and racial factors that contribute to poor outcome for patients with PSC (Lee et al., 2021), and PSC patients have a significantly increased risk of developing hepatobiliary and colorectal cancers (Gulamhusein et al., 2016). Although there are no widely used molecularly targeted therapeutic strategies available to patients with PSC (Gochanour et al., 2020; Tabibian et al., 2018), recent advances have led to a range of pharmacological agents that could be used in PSC treatment (Assis and Bowlus, 2023). Many mammalian models of PSC and liver cholestasis, which is a build-up of bile in the liver, have been generated. These include *Mdr2*^−/−^ (*Abcb4*^−/−^), *Cftr*^−/−^ and *fch/fch* knockout mice, chemically induced rat and mouse models and infection-based models (Fickert et al., 2014; Pollheimer et al., 2011). However, none of these fully recapitulate the wide-spanning and multisystem hallmarks of human PSC. This lack of available tools has substantially hampered translational research (Tabibian et al., 2018). PSC therefore remains associated with poor prognosis, often progressing to end-stage liver disease that necessitates liver resection or transplantation (Khungar and Goldberg, 2016).

A number of factors may contribute towards PSC pathogenesis. Genome-wide association studies (GWAS) have found that offspring and siblings of PSC patients have a significantly increased risk of developing PSC (Bergquist et al., 2008). Despite numerous large-scale genetic investigations looking for causal mutant variants in potential ‘PSC genes’ (Alberts et al., 2018; Karlsen et al., 2010; Melum et al., 2011; Srivastava et al., 2012), none have been conclusively determined. Instead, a combination of genetic susceptibility and other contributing factors, such as gut microbiota composition or chronic hepatic injury, may lead to PSC (Tabibian et al., 2014b; Kummen et al., 2017; Bajer et al., 2017; Torres et al., 2018). Recent data also indicate that NRAS-associated cholangiocyte senescence is a pathophysiologically important feature of PSC, and that NRAS inhibition could have therapeutic potential for patients (Tabibian et al., 2014a). Interestingly, induced senescence in mice did not recapitulate the human PSC phenotype; however, it did impair the ability of the liver to function and regenerate normally (Ferreira-Gonzalez et al., 2018).

Because NOTCH signalling is integral to the development of mammalian bile ducts (Boulter et al., 2012; Zong et al., 2009), re-activation or alterations in this pathway could contribute to the pathogenesis of PSC. Transcriptional analysis of whole-liver biopsies from PSC patients and non-affected individuals revealed no significant differences in the expression levels of *NOTCH1*, *NOTCH2* or *NOTCH4*; however, *NOTCH3* mRNA was consistently elevated in PSC whole-liver lysates (Nijjar et al., 2001). Furthermore, the NOTCH3 protein localizes to the plasma membrane in proliferating ductules, whereas in intact/mature bile ducts of PSC patients there was a total loss of expression of any of the four NOTCH receptors, including NOTCH3 (Nijjar et al., 2001). Interestingly, laser-capture microdissection of PSC ducts demonstrated that *NOTCH1* and *NOTCH2* mRNA levels were significantly upregulated in ductular cells of PSC patients compared to those in patients with fibrosis due to hepatitis C virus infection (Boulter et al., 2012), suggesting that, across PSC patients, a variety of NOTCH receptors promote pathological ductular growth.

Although the presence of NOTCH receptors indicates that NOTCH signalling may play some role in PSC, pathway activation requires the interaction of cognate ligands. Changes in the expression and distribution of the five canonical DSL ligands – JAG1, JAG2, DLL1, DLL3 and DLL4, as well as of downstream pathway modulators Deltex (DTX) and Lunatic Fringe (LFNG), and effector HES1, between PSC and healthy liver samples have been described (Nijjar et al., 2002). In PSC tissue, HES1 was expressed in all of the liver cell populations examined; however, alternative functional studies found that downregulation of HES1 in murine HPCs is crucial for PDX1-dependent cholangiocyte proliferation and biliary tree regrowth in response to cholestatic injury (Marzioni et al., 2013). Furthermore, the expression of alternative downstream effectors of NOTCH signalling, such as *HEY1* and *HEYL*, is increased in BECs from PSC patients and mouse models of this disease (Boulter et al., 2012), indicating that the NOTCH signalling response in BECs could diversify downstream of receptor activation.

As NOTCH signalling in PSC BECs requires NOTCH-activating ligands in close proximity to BECs, the question arises as to which cells within the so-called ductular reaction, a combination of BECs and their immune-stromal microenvironment, provide these ligands. In addition to cell-autonomous expression of JAG1 by BECs and BEC-adjacent myofibroblasts (Boulter et al., 2012; Spee et al., 2010), M1-polarized macrophages derived from mouse bone marrow were found to promote the self-renewing capability of HPCs via JAG1, potentially linking the pro-inflammatory processes in PSC to positive regulation of NOTCH signalling and HPC proliferation (Li et al., 2018). Pharmacological inhibition of NOTCH signalling decreased differentiation of HPCs into cholangiocytes, with an associated reduction of cholangiocyte-specific biomarkers that include Cytokeratin19 (KRT19), SOX9 and EPCAM, and a decrease in ductular fibrosis (Boulter et al., 2012; Zhang et al., 2016). Together, these data suggest that understanding how to disrupt NOTCH holds potential for the treatment of patients with PSC.

### Primary biliary cholangitis

Primary biliary cholangitis (PBC; previously referred to as primary biliary cirrhosis) is a progressive and chronic autoimmune disease characterized by irreversible damage to the small intrahepatic bile ducts (reviewed in Carey et al., 2015). Persistent biliary damage results in cholestasis and biliary fibrosis (Kumagi and Heathcote, 2008). In contrast to PSC, PBC has a strong female predominance and a median age of diagnosis between the sixth and seventh decade of life (Gershwin et al., 2005; Lindor et al., 2009). The current U.S. Food and Drug Administration (FDA)-approved standard-of-care therapy for PBC is treatment with ursodeoxycholic acid (UDCA) or obeticholic acid, both of which increase the secretion of other bile acids, eliminate toxic substances from hepatocytes and stimulate the secretion of a bicarbonate-rich fluid from cholangiocytes, thereby reducing cholestasis (Poupon, 2012). Although UDCA significantly improves the rate of transplant-free survival in the majority of PBC patients and delays the progression of hepatic fibrosis in early-stage disease, ∼40% of patients do not have an adequate biochemical response to these treatments (Barba Bernal et al., 2023; Nevens et al., 2023) and therefore do not benefit from a decrease in risk of liver transplantation or death (Carey et al., 2015; Corpechot et al., 2005).

The aetiopathogenesis of PBC remains largely unknown and may arise from a combination of genetic predisposition, immunological dysfunction and environmental triggers (Gershwin et al., 2005; Selmi et al., 2004). Fittingly, numerous GWAS datasets have identified risk loci for PBC in innate and adaptive immunity mediators (reviewed in Gulamhusein et al., 2015).

Using micro-dissection to isolate areas of ductular reaction from human PBC samples, Spee et al. demonstrated that *NOTCH2*, *JAG1* and *JAG2* mRNA expression was significantly elevated in PBC livers in a manner analogous to that previously seen in bile duct development and in PSC (Spee et al., 2010; Nijjar et al., 2001, 2002). Surprisingly, perhaps, NOTCH1 protein expression was totally undetectable in the bile ducts of PBC livers compared to low-level expression in unaffected samples (Nijjar et al., 2001), implying that NOTCH2 is the principal receptor for which expression is altered in PBC, and its cognate ligands are most likely those of the Jagged family (JAG1 and JAG2). Importantly, GWAS of 2000 PBC patients identified a susceptibility locus on chromosome 3q13.33, which indicates that increases in the expression of *POGLUT1*, which encodes a protein-*O*-glucosyltransferase that modifies NOTCH receptors, may be an effector of PBC pathogenesis *in vivo* (Hitomi et al., 2019). This is the first study to suggest that PBC may arise from abnormal regulation of NOTCH signalling, namely a pathogenic increase in glycosylation of NOTCH receptors. Increased POGLUT1 activity may result in a change in ligand affinity of various NOTCH receptors and/or overactivation of the NOTCH signalling pathway. It is therefore possible that PBC could be a result, at least in part, of impaired regulation of NOTCH signalling.

A number of rodent models have been generated to investigate PBC pathophysiology (Katsumi et al., 2015; Pollheimer and Fickert, 2015). In mouse models of cholestatic liver fibrosis, activation of NOTCH signalling is required for HPC differentiation into BECs, and the pharmacological inhibition of NOTCH receptor cleavage significantly decreases bile duct proliferation and reduces hepatic fibrosis (Mu et al., 2017; Zhang et al., 2016). Furthermore, in a murine cholestatic liver fibrosis model generated by bile duct ligation, mRNA and protein expression of NOTCH2, NOTCH3, NOTCH4, JAG1, JAG2 and RBPJκ, but not of NOTCH1, were each increased following ductular injury, although the exact cell types involved in the respective upregulation of these NOTCH signalling components were not determined (Zhang et al., 2016).

### Polycystic liver disease

Polycystic liver diseases (PLDs) are a group of inherited disorders that result from malformation of the embryonic ductal plate and are characterized by the formation of numerous cysts throughout the liver (Zhang et al., 2020a). Hepatic cystogenesis involves the abnormal extracellular secretion of fluid, cholangiocyte hyperproliferation and altered cell–matrix interactions (Banales et al., 2008; Perugorria et al., 2014), and is caused by inactivating mutations in a number of genes that regulate the formation of the primary cilia (Masyuk et al., 2009). BECs have a single cilium that protrudes into the biliary lumen and has crucial functions in mechano-, chemo- and osmo-sensing (Masyuk et al., 2006, 2008).

Autosomal-dominant polycystic kidney disease (ADPKD) is the most common form of PLD, with a prevalence of ∼1 in 1000 in the general population (Lanktree et al., 2018). ADPKD is characterized by multiple cysts growing throughout the kidney, liver and pancreas (Cornec-Le Gall et al., 2019). Autosomal-dominant polycystic liver disease (ADPLD) is a less common iteration of PLD, with cystic presentation only in the liver (Peces et al., 2005) and a prevalence of ∼1 in 10,000 individuals (Suwabe et al., 2020). Other rare forms of fibropolycystic disease that predominantly affect the liver include congenital hepatic fibrosis (Zhu et al., 2020), Caroli disease (Yonem and Bayraktar, 2007) and autosomal-recessive polycystic kidney disease (ARPKD) (Zerres et al., 2003). Patients with congenital hepatic fibrosis, Caroli disease and ARPKD generally have more severe presentations than those with ADPKD or ADPLD, characterized by excessive peribiliary fibrosis that can result in cholangitis and severe portal hypertension (Yonem and Bayraktar, 2007; Zerres et al., 2003; Zhu et al., 2020). Notably, patients with congenital hepatic fibrosis or Caroli disease have an increased risk of developing cholangiocarcinoma, discussed more below (Baghbanian et al., 2013; Nakanuma et al., 2010).

Genetic defects and cell signalling alterations have both been implicated in PLD pathogenesis. The most common causative mutations occur in the polycystin-encoding cilia genes *PKD1* and *PKD2* (Johnson and Gabow, 1997; Robinson et al., 2012). Mutations in *PKHD1*, a gene encoding fibrocystin, which is predominantly a cilium-associated protein involved in bile duct differentiation and tubulogenesis, are associated with ARPKD (Bergmann, 2017). *PKHD1* mutations have also been the primary genetic defects identified in patients with congenital hepatic fibrosis and Caroli disease (Gunay-Aygun et al., 2010; Hao et al., 2014). Membrane-bound fibrocystin undergoes NOTCH-like proteolytic processing, with translocation of a cleaved C-terminal fragment to the nucleus, where it regulates the transcription of target genes (Kaimori et al., 2007). However, the mechanism of proteolytic cleavage and the exact biological outcomes of this process are poorly understood. ADPLD has been associated with mutations in multiple genes (Drenth et al., 2003; Davila et al., 2004; Cnossen et al., 2014; Porath et al., 2016; Besse et al., 2017). Additionally, liver cysts arise in a range of syndromic diseases such as Sensenbrenner syndrome, in which WDR35 is mutated and cilia are altered (Waddell et al., 2022)*.* Further aspects of the molecular pathology of PLD have recently been reviewed (Olaizola et al., 2022).

In tissues from Caroli disease and PLD patients, NOTCH receptors and ligands are expressed by cystic epithelial cells in which HES1 accumulates in the nucleus (Takahashi et al., 2021). NOTCH signalling is important in maintaining the length of primary cilia (Lopes et al., 2010), and NOTCH signal transduction is directly affected by morphogenic changes to the cilia (Ezratty et al., 2011). Because PLDs are associated with mutations in genes that encode essential cilia-associated proteins, it is plausible that aberrant NOTCH signalling may facilitate cystogenesis. Indeed, several studies have begun to question the role of NOTCH signalling in PLD pathogenesis. Constitutive activation of the NOTCH2 ICD during murine development leads to ectopic biliary growth in areas outside of the ductal plate, ultimately leading to hepatobiliary cysts that are filled with bile or blood (Jeliazkova et al., 2013). In this model, NOTCH2 ICD-expressing cells lined tubular, tubular–cystic and microcystic structures, and expressed biliary lineage markers such as HNF1β, SOX9 and E-cadherin (CDH1) (Jeliazkova et al., 2013). Analysis of livers from ADPKD patients and autosomal-dominant and autosomal-recessive *Cpk*-mutant mouse models of polycystic kidney disease, which develop cysts as a result of mutations in cystin-1, revealed that, of the various NOTCH pathway members, NOTCH3 was consistently upregulated in cyst-lining epithelial cells (Idowu et al., 2018). Furthermore, pharmacological inhibition of NOTCH signalling significantly decreased NOTCH3 activation and overall incidence of cystogenesis in mice, as well as the growth of cultured cystic epithelial cells isolated from a patient with ADPKD (Idowu et al., 2018). Moreover, Furubo and colleagues used a polycystic kidney rat model to study changes in the NOTCH and HIPPO signalling pathways in Caroli disease specifically (Furubo et al., 2013). The authors observed expression of all four NOTCH receptors in the portal tracts and bile ducts, and an increase in the numbers of JAG1-expressing periductal and periportal myofibroblasts. In these rats, increased JAG1 expression in myofibroblasts was accompanied by elevated numbers of neighbouring NOTCH2-positive and HES1-positive BECs, which may be an important signalling relationship characteristic of Caroli disease patients (Furubo et al., 2013).

## Activation of NOTCH signalling as a driver of cholangiocarcinoma

**Fig. 4.**
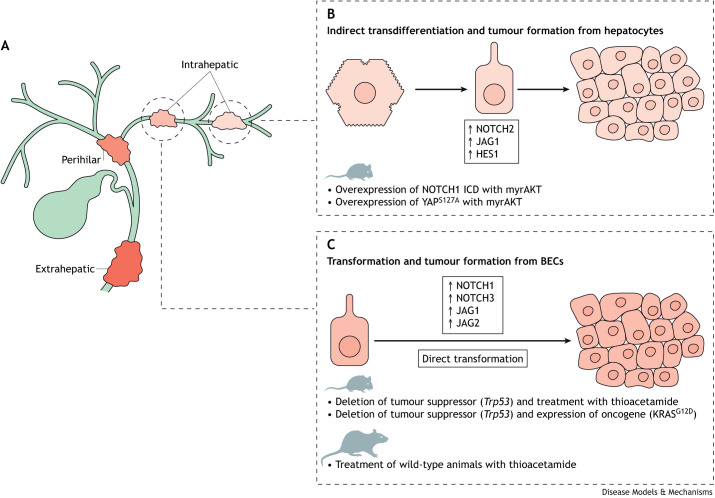
**NOTCH signalling is a central driver of intrahepatic cholangiocarcinoma in rodent models.** (A) Within the bile duct, cholangiocarcinoma arises in discreet anatomical locations and is thus characterized as extrahepatic, perihilar or intrahepatic. (B) Intrahepatic cholangiocarcinoma can arise from hepatocytes in the mouse liver. This process requires the expression of oncogenes such as NOTCH1 intracellular domain (ICD) or mutant YAP along with constitutively active myristoylated AKT (myrAKT). This results in lineage conversion of hepatocytes into a BEC-like phenotype and drives tumour formation, which requires NOTCH signalling. (C) Intrahepatic cholangiocarcinoma can also arise from BECs through direct transformation of the biliary epithelium, either through mutations or through chronic injury during which cells upregulate components of the NOTCH signalling pathway.

Cholangiocarcinoma is a highly aggressive and genetically complex cancer of the bile ducts. Cholangiocarcinoma may arise from bile ducts within the liver (intrahepatic), at the confluence of bile ducts leaving the liver (perihilar) or from the bile ducts outside the liver (distal cholangiocarcinoma) (Banales et al., 2020) (Fig. 4A). Cholangiocarcinoma typically presents late in life at an advanced stage, and, as a result, only one-third of patients are eligible for a potentially curable surgical resection (Forner et al., 2019). Additionally, the relapse rate is high, with∼50% of resected patients presenting with recurrent disease. The 5-year survival rate of a patient with cholangiocarcinoma has remained static in the United Kingdom at ∼1 in 20 since the late 1970s (Alabraba et al., 2019), with global survival varying depending on country and subtype (Steele et al., 2018; Vithayathil and Khan, 2022). The standard of care for cholangiocarcinoma is combination chemotherapy, normally gemcitabine and cisplatin, which typically extends life by a number of months (Valle et al., 2010). Recent advances have seen treatment of cholangiocarcinoma with folinic acid (leucovorin), fluorouracil and oxaliplatin (FOLFOX) in the second-line setting (Lamarca et al., 2021), as well as a number of targeted therapies. Indeed, precision medicine approaches in cholangiocarcinoma have rapidly expanded in recent years, and pharmacological targeting of FGFR2 fusions (Goyal et al., 2023), IDH1 neomorphic mutants (Zhu et al., 2021), and mutations in kinases including BRAF, HER2 (ERBB2) and NTRK (Doebele et al., 2020; Javle et al., 2021; Subbiah et al., 2020) have shown clinical promise. Moreover, although initial results with immunotherapy in cholangiocarcinoma were disappointing (Piha-Paul et al., 2020), the combination of immunotherapy with chemotherapy or targeted therapy is showing increased clinical potential (Oh et al., 2022; Zhang et al., 2023).

Patients with highly regenerative, fibro-inflammatory biliary diseases, including PSC and chronic infection with liver fluke (*Opisthorchis viverrini*), progress to cholangiocarcinoma more commonly than do patients with other fibrotic liver diseases, such as chronic infection with hepatitis virus or chronic alcohol consumption (Barner-Rasmussen et al., 2020; Karlsen et al., 2017). This diseased and regenerating pre-neoplastic state seems to be permissive to malignancy, and cells and signals from the regenerative microenvironment are essential for cancer growth (Boulter et al., 2015; Clapéron et al., 2013; Zhang et al., 2020b). In cholangiocarcinoma, a subset of patients have high levels of NOTCH signalling activity and are therefore potentially amenable to treatment with pharmacological NOTCH inhibitors, particularly those preventing NOTCH receptor cleavage (O'Rourke et al., 2020; Vanaroj et al., 2022).

Rodent models of cholangiocarcinoma have proven valuable in understanding the pathological processes that underpin its initiation and progression. In patient tissue, both *NOTCH1* and *NOTCH3* are overexpressed in tumour cells compared to adjacent non-cancerous bile ducts, and, by contrast, expression of *NOTCH2*, which is critical for ductular formation in the embryo, does not appear to appreciably change between pre-neoplastic and neoplastic states (Guest et al., 2016; O'Rourke et al., 2020). Early work concentrating on the role of NOTCH1 in cholangiocarcinoma demonstrated its role in the proliferation and migration of cholangiocarcinoma cells (Zender et al., 2013; Zhou et al., 2013), and in mediating their chemosensitivity through the regulation of chemoefflux transporters (Wu et al., 2014). Moreover, ectopic expression of the human NOTCH1 ICD, in combination with the expression of constitutively active myristoylated AKT (myrAKT), induces the formation of intrahepatic cholangiocarcinoma in mice in the absence of underlying disease (Fan et al., 2012) by causing hepatocytes to undergo hepatocyte-to-cholangiocyte conversion (Sekiya and Suzuki, 2012) (Fig. 4B). Subsequent studies demonstrated that this conversion required both the NOTCH ligand JAG1 and the downstream effector HES1 (Guo et al., 2019; Huntzicker et al., 2015).

Given the reiterative use of NOTCH2 in mammalian bile duct biology, it is somewhat surprising that these early studies have identified NOTCH1 as essential for the formation of hepatocyte-derived cholangiocarcinoma (Fan et al., 2012). This was addressed in a hepatocyte-derived cholangiocarcinoma model, which was driven by the expression of oncogenic YAP (YAP^S127A^) and myrAKT and thus did not rely on the ectopic expression of the NOTCH1 ICD (Wang et al., 2018). Using this model in combination with specific deletion of *Notch1* or *Notch2* genes, Wang et al. clearly demonstrated that there is a functional requirement for NOTCH2 in cholangiocarcinoma, because the deletion of NOTCH2 in this model resulted in hepatocellular carcinoma. Therefore, in the context of cancer, signalling through the NOTCH2 receptor is required for linage conversion of biliary cells and cholangiocarcinogenesis (Wang et al., 2018). NOTCH signalling activation in hepatocytes drives both SOX9 and YAP1 expression; however, only YAP1–TEAD signalling is essential for the NOTCH-dependent formation of malignant BECs from hepatocytes. Through DNMT1-mediated chromatin remodelling, YAP suppresses the hepatocyte lineage programme and promotes the development of cholangiocarcinoma (Hu et al., 2022).

Although murine models of hepatocyte-derived cholangiocarcinoma have provided crucial insights into the pathogenic processes that underpin the growth, there is relatively little evidence in human studies that this cancer typically arises from hepatocytes. Histologically and anatomically, cholangiocarcinoma seems to arise from BECs that comprise the bile duct. A number of studies have addressed whether there is a role for NOTCH signalling in cholangiocarcinoma arising from the bile duct, where cells are not required to undergo lineage conversion prior to oncogenesis. Cholangiocarcinoma can be generated from murine BECs using classical Cre/LoxP-mediated lineage-specific deletion of *Trp53* alone (Guest et al., 2014), or in tandem with either oncogenic KRAS^G12D^ (Hill et al., 2018) or *Pten* deletion (Younger et al., 2022). In these models, the NOTCH signalling pathway is constitutively activated (Fig. 4C), and NOTCH1 and NOTCH3 are highly expressed (O'Rourke et al., 2020; Zender et al., 2013). In a different murine model in which the NOTCH1 ICD is expressed in the neonatal liver, cholangiocarcinoma initiation and growth promote the transcriptional regulation of Cyclin-E (CCNE1) (Zender et al., 2013). In a separate study, genetic overexpression of NOTCH1 in the biliary epithelium accelerated KRAS^G12D^-induced cholangiocarcinoma though its canonical effector, HES1 (Matsumori et al., 2020). The role of NOTCH3 appears to be different from that of NOTCH1, and rather than regulating cell proliferation through a CSL/RBPJκ-dependent mechanism, NOTCH3 potentiates cell proliferation through activation of mTOR. Genetic deletion of *Notch3* in a mouse model of cholangiocarcinoma reduced tumour size and frequency, suggesting that NOTCH signalling, through NOTCH3 at least, is required for tumour initiation. Further *in vivo* studies suggest that NOTCH1 and NOTCH3 inhibition reduces cancer cell proliferation and tumour size (Guest et al., 2016; Kitchen et al., 2020).

In the clinical setting, identifying cholangiocarcinoma patients with activated NOTCH signalling signatures in their tumours has clear prognostic value, particularly if patients can be triaged to receive NOTCH inhibition therapies. There are a number of NOTCH pathway inhibitors in pre-clinical and clinical trials. Although none of these have been specifically developed with cholangiocarcinoma in mind, several inhibitors are being tested in solid malignancies. The majority are γ-secretase inhibitors (GSIs), which inhibit S3 cleavage, thereby preventing the processing of the NOTCH ICD. These include RO4929097, LY900009 and crenigacestat (LY3039478), the latter of which might also have some selectivity for inhibiting the processing of NOTCH1. In addition to GSIs, newer classes of NOTCH therapeutics are being explored. ADAM inhibitors that inhibit S2 cleavage of NOTCH receptors, such as GI254023X, as well as targeted receptor-blocking antibodies that interfere with receptor–ligand binding, such as OMP-52M51 (brontictuzumab) (Ferrarotto et al., 2018), OMP-59R5 (tarextumab) (Smith et al., 2019) and OMP-21M18 (demcizumab) (Smith et al., 2014), are being trialled (Huntzicker et al., 2015; Teodorczyk and Schmidt, 2014).

## Modulation of NOTCH signalling in biliary disease – opportunities and pitfalls in translation

Many of the steps of NOTCH signalling (Fig. 1) are amenable to modulation by biochemical agents or small molecules. First, blocking antibodies are capable of binding specific NOTCH receptors (Falk et al., 2012; Wu et al., 2010) or DSL ligands (El Kaffas et al., 2014; Liu et al., 2011) to prevent ligand binding. Metalloprotease inhibitors such as TMI-005 (apratastat), which specifically inhibits ADAM17/TACE (Moss et al., 2008), and INCB3619, which inhibits both ADAM10 and ADAM17/TACE (Fridman et al., 2007), prevent S2 proteolysis of NOTCH receptors, downregulating signalling activity following ligand–receptor binding. Clinically, ADAM-specific inhibition has led to off-target affects and liver toxicity (Edwards et al., 2008), most likely due to ADAM metalloproteases cleaving a large number of substrates, including tumour necrosis factor (TNF)-α (TNF) (Black et al., 1997; Moss et al., 1997). By far the most commonly used chemical modulators of NOTCH signalling are GSIs (Andersson and Lendahl, 2014; Olsauskas-Kuprys et al., 2013), which, as we discussed previously, prevent S3 proteolysis and release of the NOTCH ICD. GSIs have been used in several clinical trials to attenuate the effects of NOTCH gain-of-function mutations in cancer, with largely positive results (Geling et al., 2002; O'Rourke et al., 2020; Real et al., 2009). However, whether these will have a broad applicability to solid cancers, such as cholangiocarcinoma, remains to be seen.

## Conclusions

ALGS was defined clinically in 1975 as a pleiotropic syndrome that resulted in failure of bile duct ontogeny, attributed to causal mutant variants in *JAG1* or *NOTCH2*. Since that discovery, it has become increasingly clear that activation of NOTCH signalling regulates bile duct biology throughout life, not only being required for progenitor-mediated restoration of the hepatocyte-rich parenchyma, but also for BEC proliferation and cholangiocarcinoma growth. BECs, in both the normal wild-type context and once they are transformed in cancer, are surrounded by a complex cellular and acellular microenvironment comprising inflammatory and mesenchymal cells. These supply cognate ligands to NOTCH receptors expressed on the epithelial cells. Importantly, it appears now, from a number of studies, that in biliary regeneration, disease and cancer, the reiterative deployment of NOTCH2 and JAG1 promotes BEC, HPC and cancer cell proliferation and provides a specific signalling module that could be targeted therapeutically across disease states.

Many therapies inhibit the enzymes of the NOTCH signalling pathway because it is relatively easy to develop small molecules to interfere with a specific active site. In particular, GSIs inhibit NOTCH signalling, but also elicit systemic adverse effects, which has prohibited their widespread clinical use. Specific NOTCH receptor inhibitors and blocking antibodies are in pre-clinical development and represent a much more refined approach to modulating the NOTCH signal, particularly if bivalent inhibitors that target specific ligand–receptor interactions can be identified.

In a number of cholangiopathies, the risk of developing cholangiocarcinoma is higher than average. Animal models and patient studies have demonstrated that high NOTCH signalling denotes at least a subset of patients who could respond to NOTCH inhibitor therapy. Indeed, a number of NOTCH-related inhibitors are currently in clinical trials for the treatment of solid malignancies, including cholangiocarcinoma. For these types of approaches to become more widely applicable, however, we need to understand the diverse functions of NOTCH signalling and address whether activation of the NOTCH signalling pathway is restricted to certain sub-populations of cells within the biliary tree.

The reiterative use of NOTCH signalling in bile duct development, regeneration and disease highlights the central importance of this signalling cascade in bile duct biology; however, there are still distinct gaps in our understanding. In order to intervene appropriately at specific stages within the pathway, we must first define the spatiotemporal regulation of NOTCH signalling during the onset and progression of hepatobiliary diseases and identify which patients will benefit from these types of approaches.
